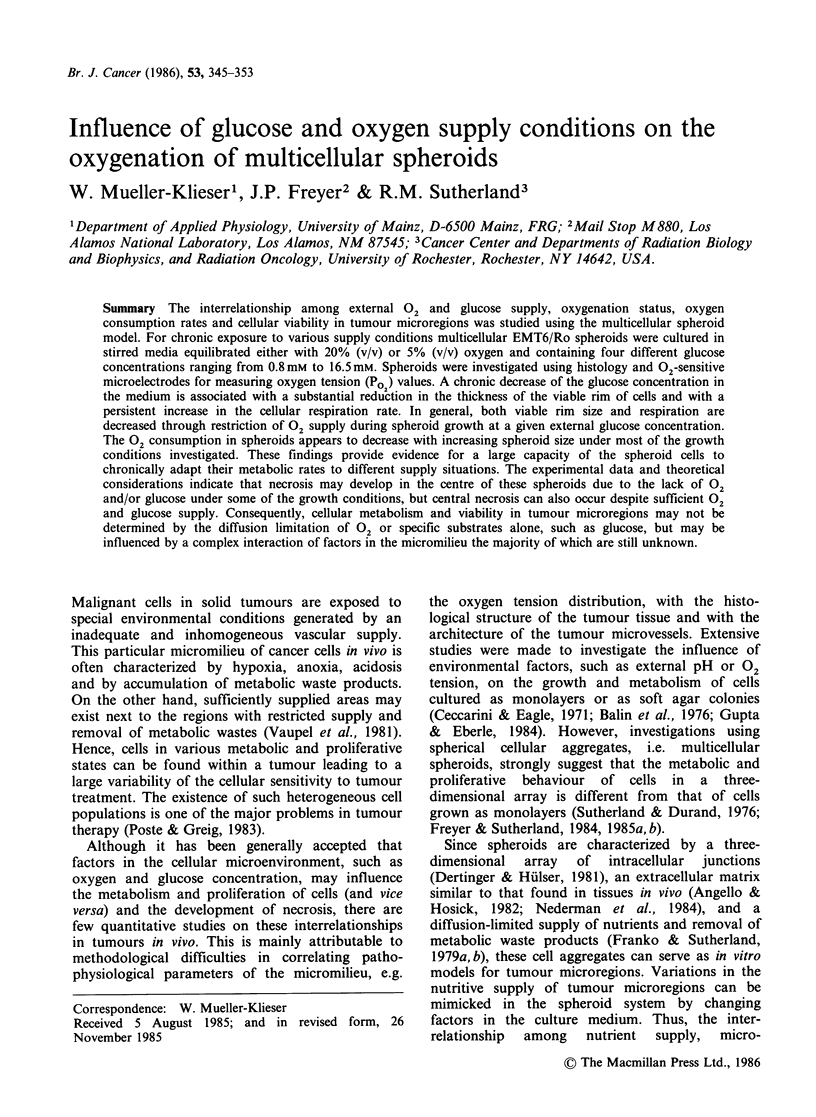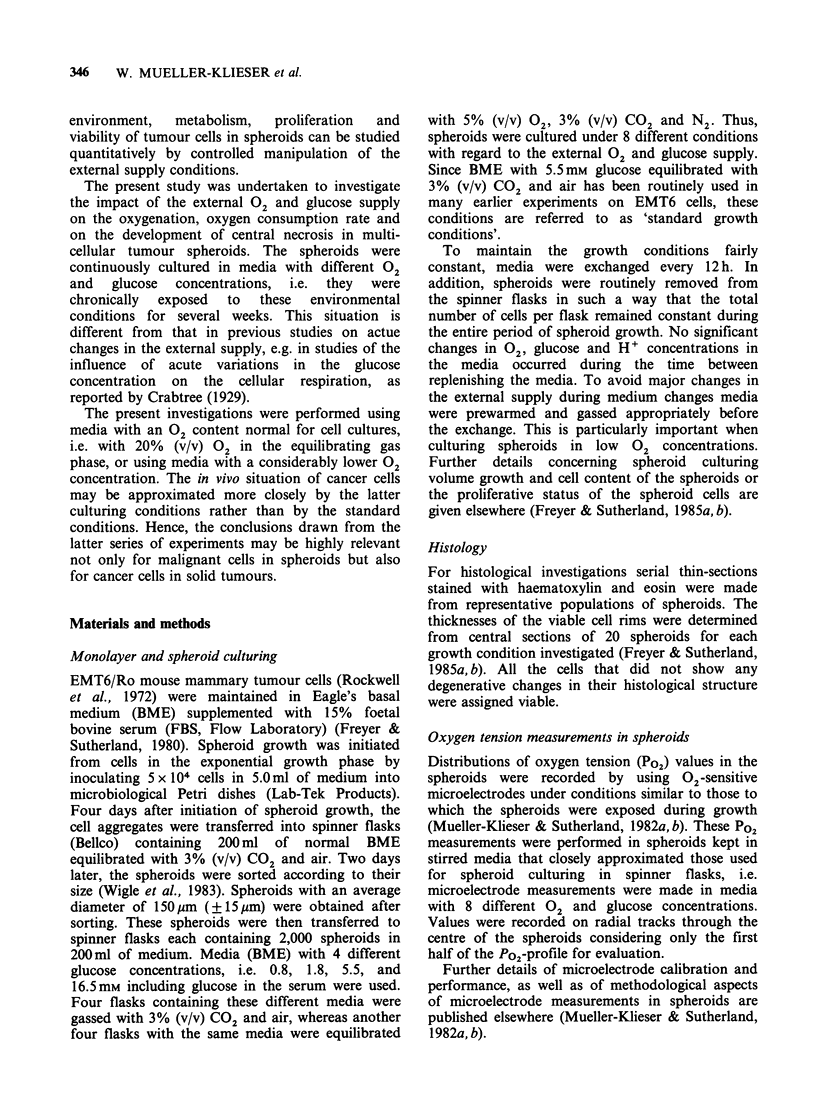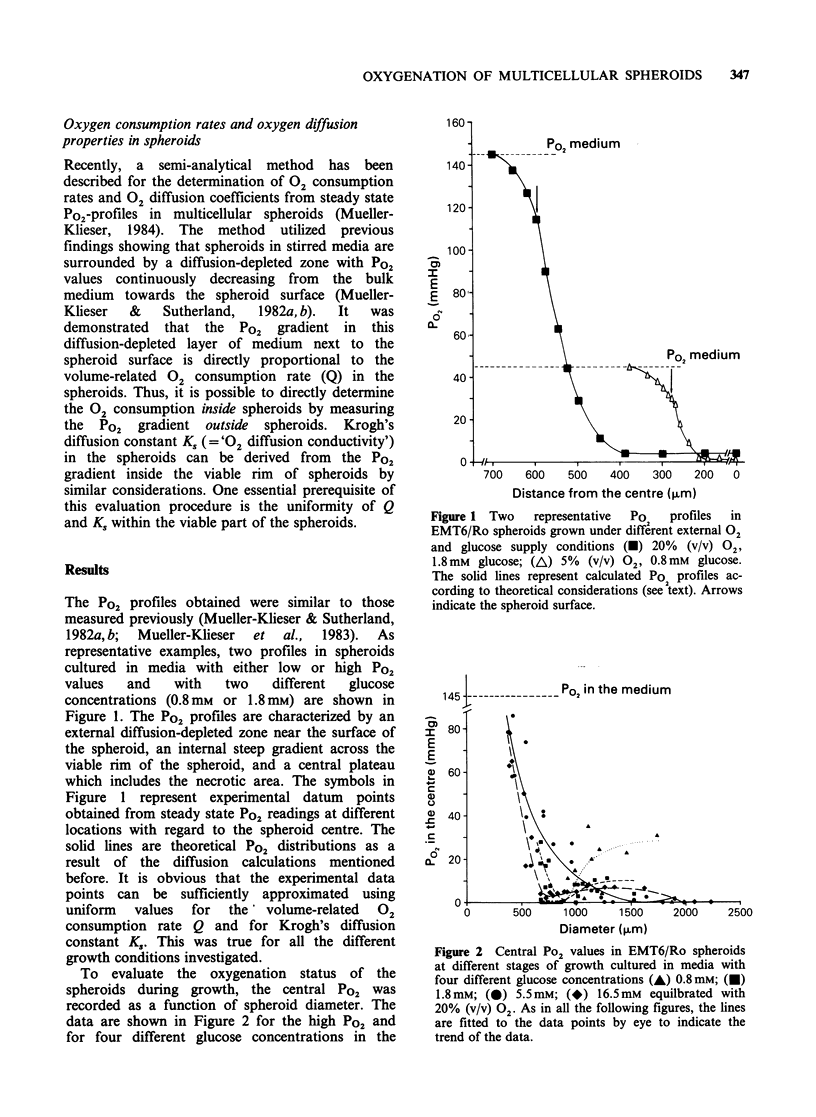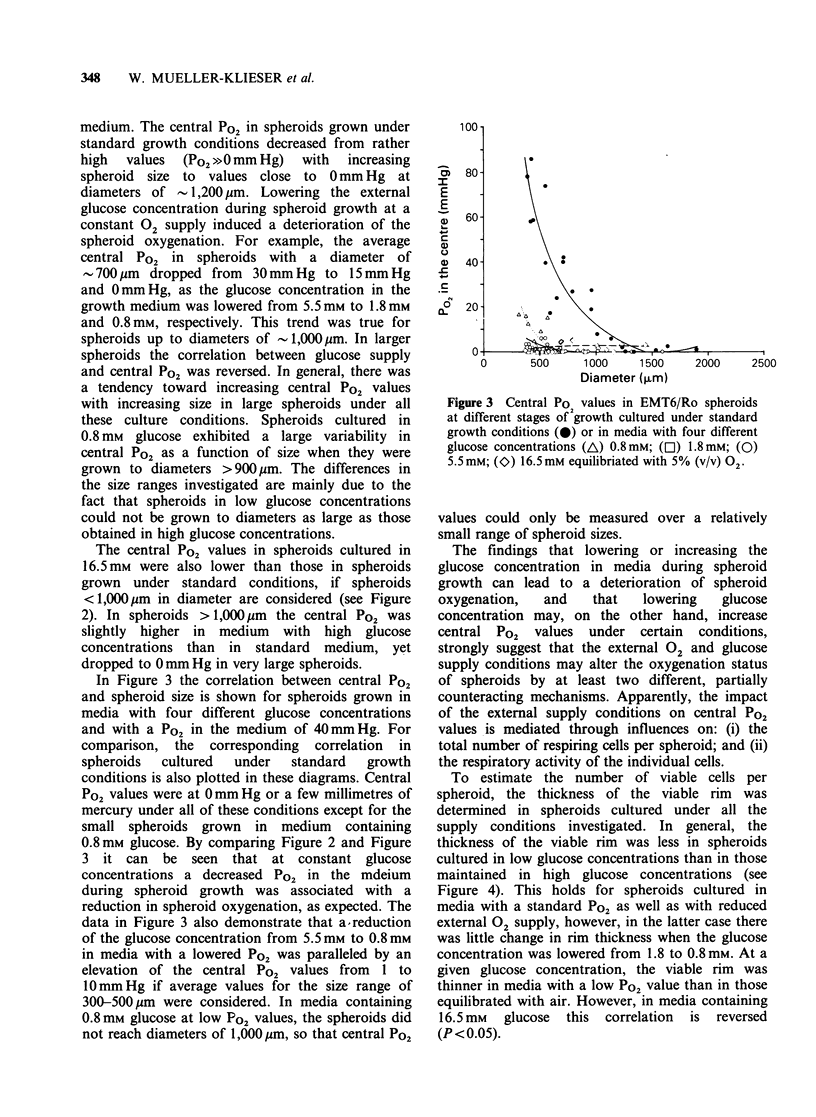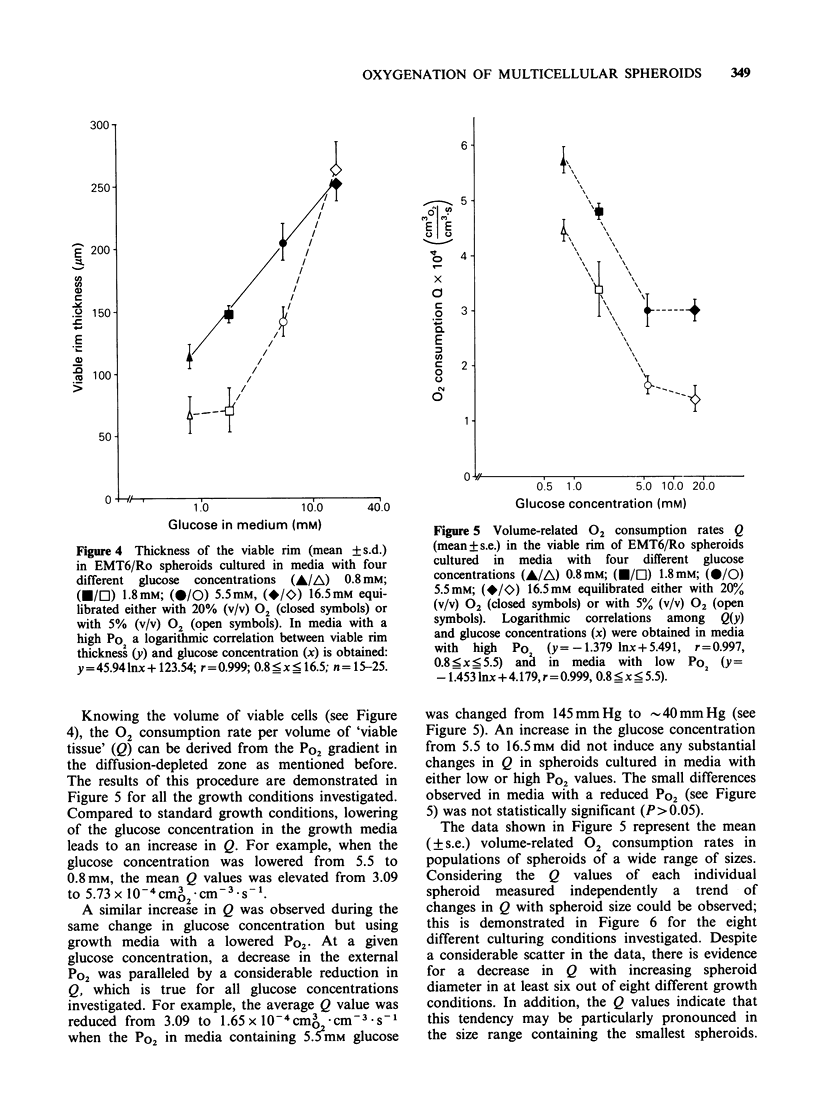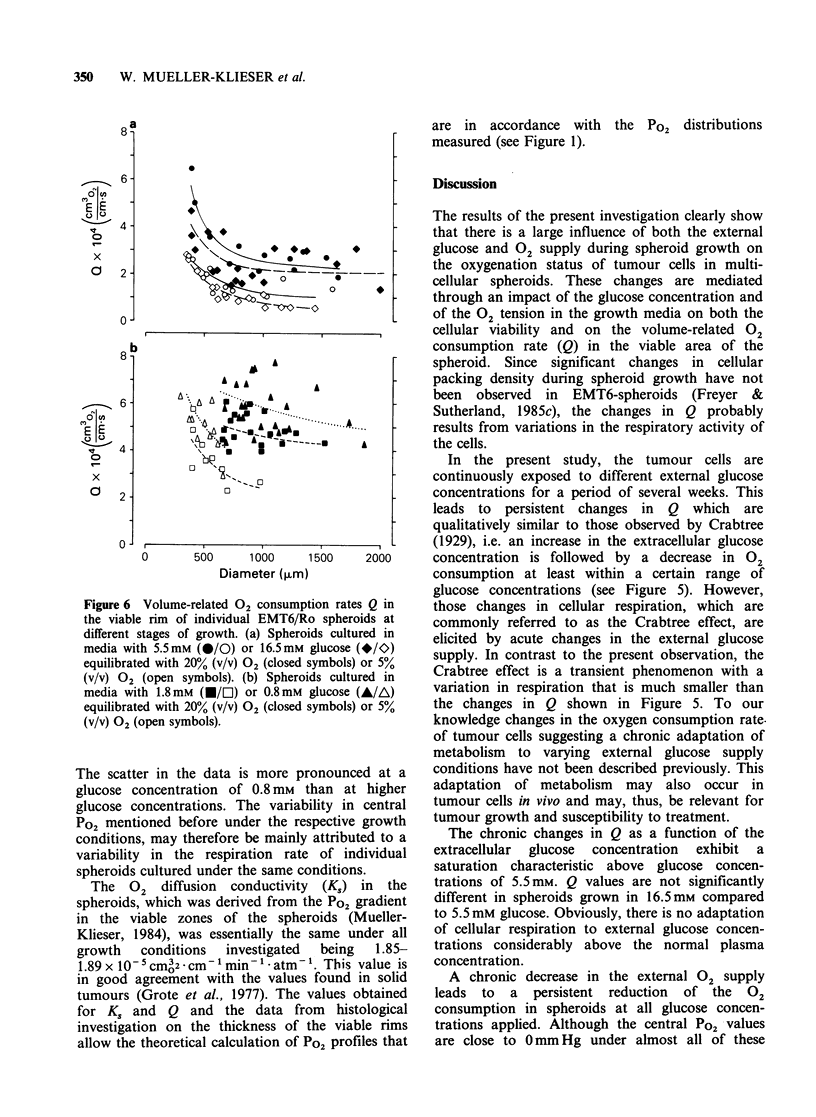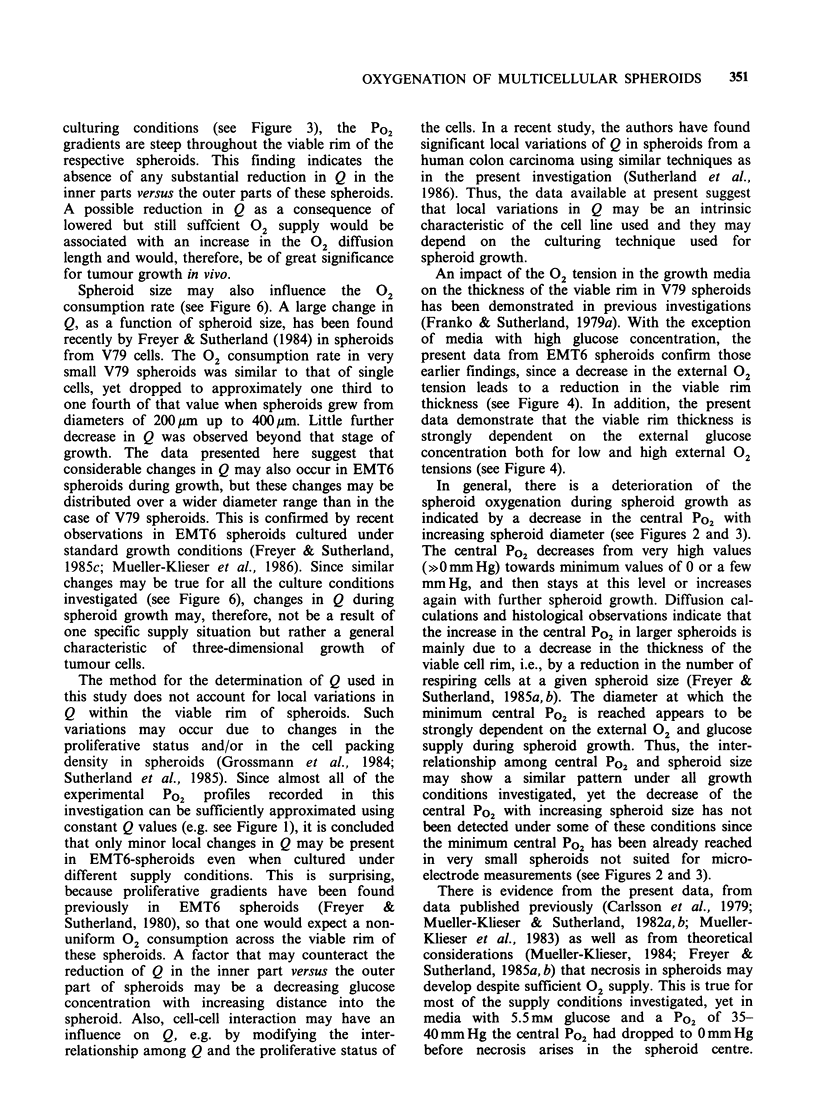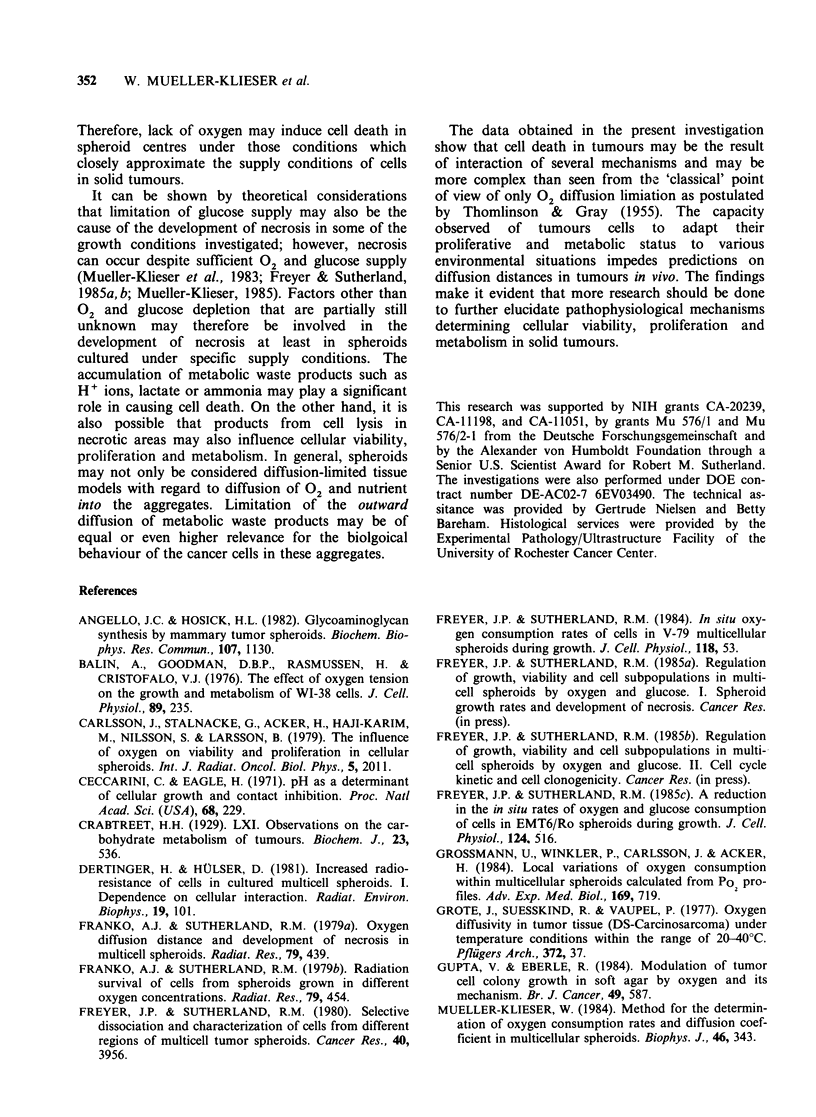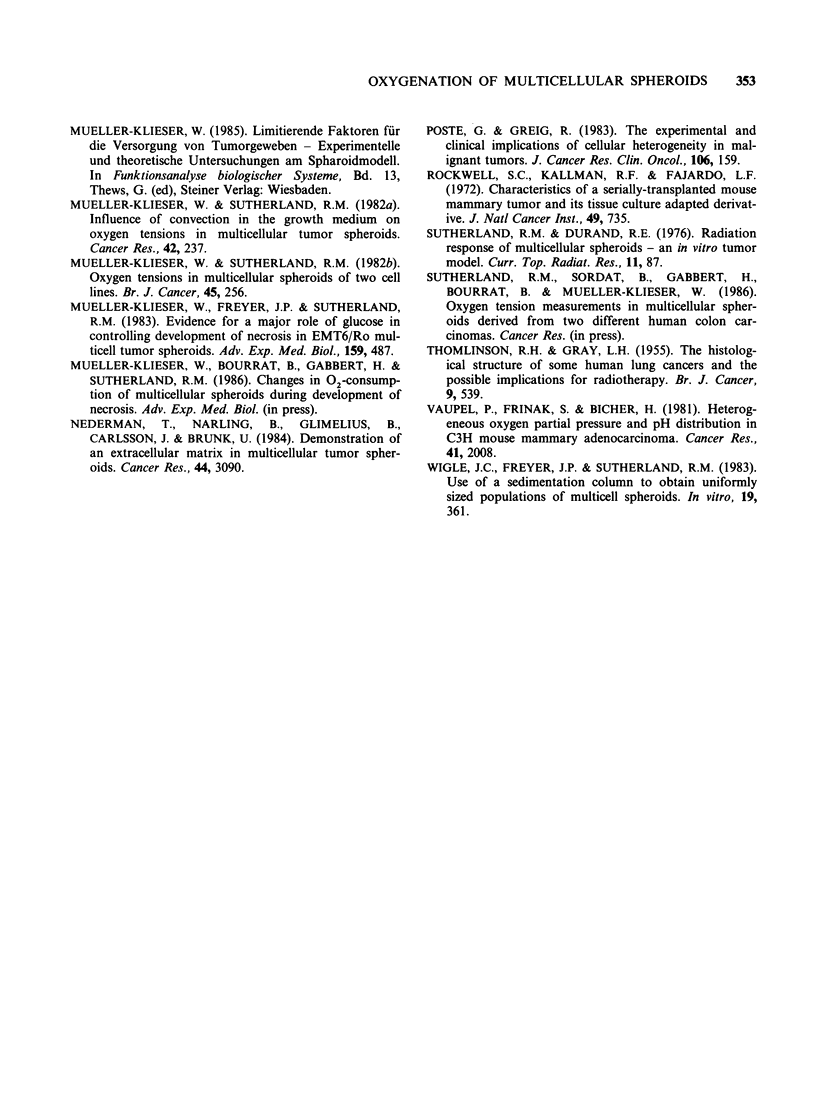# Influence of glucose and oxygen supply conditions on the oxygenation of multicellular spheroids.

**DOI:** 10.1038/bjc.1986.58

**Published:** 1986-03

**Authors:** W. Mueller-Klieser, J. P. Freyer, R. M. Sutherland

## Abstract

The interrelationship among external O2 and glucose supply, oxygenation status, oxygen consumption rates and cellular viability in tumour microregions was studied using the multicellular spheroid model. For chronic exposure to various supply conditions multicellular EMT6/Ro spheroids were cultured in stirred media equilibrated either with 20% (v/v) or 5% (v/v) oxygen and containing four different glucose concentrations ranging from 0.8 mM to 16.5 mM. Spheroids were investigated using histology and O2-sensitive microelectrodes for measuring oxygen tension (PO2) values. A chronic decrease of the glucose concentration in the medium is associated with a substantial reduction in the thickness of the viable rim of cells and with a persistent increase in the cellular respiration rate. In general, both viable rim size and respiration are decreased through restriction of O2 supply during spheroid growth at a given external glucose concentration. The O2 consumption in spheroids appears to decrease with increasing spheroid size under most of the growth conditions investigated. These findings provide evidence for a large capacity of the spheroid cells to chronically adapt their metabolic rates to different supply situations. The experimental data and theoretical considerations indicate that necrosis may develop in the centre of these spheroids due to the lack of O2 and/or glucose under some of the growth conditions, but central necrosis can also occur despite sufficient O2 and glucose supply. Consequently, cellular metabolism and viability in tumour microregions may not be determined by the diffusion limitation of O2 or specific substrates alone, such as glucose, but may be influenced by a complex interaction of factors in the micromilieu the majority of which are still unknown.


Br. J. Cancer (1986), 53, 345–353

### Influence of glucose and oxygen supply conditions on the oxygenation of multicellular spheroids

W. Mueller-Klieser[1], J.P. Freyer[2] & R.M. Sutherland[3]

[1]*Department of Applied Physiology, University of Mainz, D-6500 Mainz, FRG;* [2]*Mail Stop M880, Los Alamos National Laboratory, Los Alamos, NM 87545;* [3]*Cancer Center and Departments of Radiation Biology and Biophysics, and Radiation Oncology, University of Rochester, Rochester, NY 14642, USA.*

**Summary** The interrelationship among external $O_2$ and glucose supply, oxygenation status, oxygen consumption rates and cellular viability in tumour microregions was studied using the multicellular spheroid model. For chronic exposure to various supply conditions multicellular EMT6/Ro spheroids were cultured in stirred media equilibrated either with 20% (v/v) or 5% (v/v) oxygen and containing four different glucose concentrations ranging from 0.8 mM to 16.5 mM. Spheroids were investigated using histology and $O_2$-sensitive microelectrodes for measuring oxygen tension ($P_{O_2}$) values. A chronic decrease of the glucose concentration in the medium is associated with a substantial reduction in the thickness of the viable rim of cells and with a persistent increase in the cellular respiration rate. In general, both viable rim size and respiration are decreased through restriction of $O_2$ supply during spheroid growth at a given external glucose concentration. The $O_2$ consumption in spheroids appears to decrease with increasing spheroid size under most of the growth conditions investigated. These findings provide evidence for a large capacity of the spheroid cells to chronically adapt their metabolic rates to different supply situations. The experimental data and theoretical considerations indicate that necrosis may develop in the centre of these spheroids due to the lack of $O_2$ and/or glucose under some of the growth conditions, but central necrosis can also occur despite sufficient $O_2$ and glucose supply. Consequently, cellular metabolism and viability in tumour microregions may not be determined by the diffusion limitation of $O_2$ or specific substrates alone, such as glucose, but may be influenced by a complex interaction of factors in the micromilieu the majority of which are still unknown.

Malignant cells in solid tumours are exposed to special environmental conditions generated by an inadequate and inhomogeneous vascular supply. This particular micromilieu of cancer cells *in vivo* is often characterized by hypoxia, anoxia, acidosis and by accumulation of metabolic waste products. On the other hand, sufficiently supplied areas may exist next to the regions with restricted supply and removal of metabolic wastes (Vaupel *et al.*, 1981). Hence, cells in various metabolic and proliferative states can be found within a tumour leading to a large variability of the cellular sensitivity to tumour treatment. The existence of such heterogeneous cell populations is one of the major problems in tumour therapy (Poste & Greig, 1983).

Although it has been generally accepted that factors in the cellular microenvironment, such as oxygen and glucose concentration, may influence the metabolism and proliferation of cells (and *vice versa*) and the development of necrosis, there are few quantitative studies on these interrelationships in tumours *in vivo*. This is mainly attributable to methodological difficulties in correlating pathophysiological parameters of the micromilieu, e.g. the oxygen tension distribution, with the histological structure of the tumour tissue and with the architecture of the tumour microvessels. Extensive studies were made to investigate the influence of environmental factors, such as external pH or $O_2$ tension, on the growth and metabolism of cells cultured as monolayers or as soft agar colonies (Ceccarini & Eagle, 1971; Balin *et al.*, 1976; Gupta & Eberle, 1984). However, investigations using spherical cellular aggregates, i.e. multicellular spheroids, strongly suggest that the metabolic and proliferative behaviour of cells in a three-dimensional array is different from that of cells grown as monolayers (Sutherland & Durand, 1976; Freyer & Sutherland, 1984, 1985*a, b*).

Since spheroids are characterized by a three-dimensional array of intracellular junctions (Dertinger & Hülser, 1981), an extracellular matrix similar to that found in tissues *in vivo* (Angello & Hosick, 1982; Nederman *et al.*, 1984), and a diffusion-limited supply of nutrients and removal of metabolic waste products (Franko & Sutherland, 1979*a, b*), these cell aggregates can serve as *in vitro* models for tumour microregions. Variations in the nutritive supply of tumour microregions can be mimicked in the spheroid system by changing factors in the culture medium. Thus, the interrelationship among nutrient supply, micro-

Correspondence: W. Mueller-Klieser
Received 5 August 1985; and in revised form, 26 November 1985

© The Macmillan Press Ltd., 1986

environment, metabolism, proliferation and viability of tumour cells in spheroids can be studied quantitatively by controlled manipulation of the external supply conditions.

The present study was undertaken to investigate the impact of the external $O_2$ and glucose supply on the oxygenation, oxygen consumption rate and on the development of central necrosis in multicellular tumour spheroids. The spheroids were continuously cultured in media with different $O_2$ and glucose concentrations, i.e. they were chronically exposed to these environmental conditions for several weeks. This situation is different from that in previous studies on actue changes in the external supply, e.g. in studies of the influence of acute variations in the glucose concentration on the cellular respiration, as reported by Crabtree (1929).

The present investigations were performed using media with an $O_2$ content normal for cell cultures, i.e. with 20% (v/v) $O_2$ in the equilibrating gas phase, or using media with a considerably lower $O_2$ concentration. The *in vivo* situation of cancer cells may be approximated more closely by the latter culturing conditions rather than by the standard conditions. Hence, the conclusions drawn from the latter series of experiments may be highly relevant not only for malignant cells in spheroids but also for cancer cells in solid tumours.

### Materials and methods

### *Monolayer and spheroid culturing*

EMT6/Ro mouse mammary tumour cells (Rockwell *et al.*, 1972) were maintained in Eagle's basal medium (BME) supplemented with 15% foetal bovine serum (FBS, Flow Laboratory) (Freyer & Sutherland, 1980). Spheroid growth was initiated from cells in the exponential growth phase by inoculating $5 \times 10^4$ cells in 5.0 ml of medium into microbiological Petri dishes (Lab-Tek Products). Four days after initiation of spheroid growth, the cell aggregates were transferred into spinner flasks (Bellco) containing 200 ml of normal BME equilibrated with 3% (v/v) $CO_2$ and air. Two days later, the spheroids were sorted according to their size (Wigle *et al.*, 1983). Spheroids with an average diameter of 150 $\mu$m ($\pm$15 $\mu$m) were obtained after sorting. These spheroids were then transferred to spinner flasks each containing 2,000 spheroids in 200 ml of medium. Media (BME) with 4 different glucose concentrations, i.e. 0.8, 1.8, 5.5, and 16.5 mM including glucose in the serum were used. Four flasks containing these different media were gassed with 3% (v/v) $CO_2$ and air, whereas another four flasks with the same media were equilibrated with 5% (v/v) $O_2$, 3% (v/v) $CO_2$ and $N_2$. Thus, spheroids were cultured under 8 different conditions with regard to the external $O_2$ and glucose supply. Since BME with 5.5 mM glucose equilibrated with 3% (v/v) $CO_2$ and air has been routinely used in many earlier experiments on EMT6 cells, these conditions are referred to as 'standard growth conditions'.

To maintain the growth conditions fairly constant, media were exchanged every 12 h. In addition, spheroids were routinely removed from the spinner flasks in such a way that the total number of cells per flask remained constant during the entire period of spheroid growth. No significant changes in $O_2$, glucose and $H^+$ concentrations in the media occurred during the time between replenishing the media. To avoid major changes in the external supply during medium changes media were prewarmed and gassed appropriately before the exchange. This is particularly important when culturing spheroids in low $O_2$ concentrations. Further details concerning spheroid culturing volume growth and cell content of the spheroids or the proliferative status of the spheroid cells are given elsewhere (Freyer & Sutherland, 1985*a*, *b*).

### *Histology*

For histological investigations serial thin-sections stained with haematoxylin and eosin were made from representative populations of spheroids. The thicknesses of the viable cell rims were determined from central sections of 20 spheroids for each growth condition investigated (Freyer & Sutherland, 1985*a*, *b*). All the cells that did not show any degenerative changes in their histological structure were assigned viable.

### *Oxygen tension measurements in spheroids*

Distributions of oxygen tension ($P_{O_2}$) values in the spheroids were recorded by using $O_2$-sensitive microelectrodes under conditions similar to those to which the spheroids were exposed during growth (Mueller-Klieser & Sutherland, 1982*a*, *b*). These $P_{O_2}$ measurements were performed in spheroids kept in stirred media that closely approximated those used for spheroid culturing in spinner flasks, i.e. microelectrode measurements were made in media with 8 different $O_2$ and glucose concentrations. Values were recorded on radial tracks through the centre of the spheroids considering only the first half of the $P_{O_2}$-profile for evaluation.

Further details of microelectrode calibration and performance, as well as of methodological aspects of microelectrode measurements in spheroids are published elsewhere (Mueller-Klieser & Sutherland, 1982*a*, *b*).

*Oxygen consumption rates and oxygen diffusion properties in spheroids*

Recently, a semi-analytical method has been described for the determination of $O_2$ consumption rates and $O_2$ diffusion coefficients from steady state $P_{O_2}$-profiles in multicellular spheroids (Mueller-Klieser, 1984). The method utilized previous findings showing that spheroids in stirred media are surrounded by a diffusion-depleted zone with $P_{O_2}$ values continuously decreasing from the bulk medium towards the spheroid surface (Mueller-Klieser & Sutherland, 1982*a*, *b*). It was demonstrated that the $P_{O_2}$ gradient in this diffusion-depleted layer of medium next to the spheroid surface is directly proportional to the volume-related $O_2$ consumption rate (Q) in the spheroids. Thus, it is possible to directly determine the $O_2$ consumption *inside* spheroids by measuring the $P_{O_2}$ gradient *outside* spheroids. Krogh's diffusion constant $K_s$ (= '$O_2$ diffusion conductivity') in the spheroids can be derived from the $P_{O_2}$ gradient inside the viable rim of spheroids by similar considerations. One essential prerequisite of this evaluation procedure is the uniformity of $Q$ and $K_s$ within the viable part of the spheroids.

### Results

The $P_{O_2}$ profiles obtained were similar to those measured previously (Mueller-Klieser & Sutherland, 1982*a*, *b*; Mueller-Klieser *et al.*, 1983). As representative examples, two profiles in spheroids cultured in media with either low or high $P_{O_2}$ values and with two different glucose concentrations (0.8 mM or 1.8 mM) are shown in Figure 1. The $P_{O_2}$ profiles are characterized by an external diffusion-depleted zone near the surface of the spheroid, an internal steep gradient across the viable rim of the spheroid, and a central plateau which includes the necrotic area. The symbols in Figure 1 represent experimental datum points obtained from steady state $P_{O_2}$ readings at different locations with regard to the spheroid centre. The solid lines are theoretical $P_{O_2}$ distributions as a result of the diffusion calculations mentioned before. It is obvious that the experimental data points can be sufficiently approximated using uniform values for the volume-related $O_2$ consumption rate $Q$ and for Krogh's diffusion constant $K_s$. This was true for all the different growth conditions investigated.

To evaluate the oxygenation status of the spheroids during growth, the central $P_{O_2}$ was recorded as a function of spheroid diameter. The data are shown in Figure 2 for the high $P_{O_2}$ and for four different glucose concentrations in the

**Figure 1** Two representative $P_{O_2}$ profiles in EMT6/Ro spheroids grown under different external $O_2$ and glucose supply conditions (■) 20% (v/v) $O_2$, 1.8 mM glucose; (△) 5% (v/v) $O_2$, 0.8 mM glucose. The solid lines represent calculated $P_{O_2}$ profiles according to theoretical considerations (see text). Arrows indicate the spheroid surface.

**Figure 2** Central $P_{O_2}$ values in EMT6/Ro spheroids at different stages of growth cultured in media with four different glucose concentrations (▲) 0.8 mM; (■) 1.8 mM; (●) 5.5 mM; (◆) 16.5 mM equilbrated with 20% (v/v) $O_2$. As in all the following figures, the lines are fitted to the data points by eye to indicate the trend of the data.

medium. The central $P_{O_2}$ in spheroids grown under standard growth conditions decreased from rather high values ($P_{O_2} \gg 0$ mm Hg) with increasing spheroid size to values close to 0 mm Hg at diameters of ~1,200 μm. Lowering the external glucose concentration during spheroid growth at a constant $O_2$ supply induced a deterioration of the spheroid oxygenation. For example, the average central $P_{O_2}$ in spheroids with a diameter of ~700 μm dropped from 30 mm Hg to 15 mm Hg and 0 mm Hg, as the glucose concentration in the growth medium was lowered from 5.5 mM to 1.8 mM and 0.8 mM, respectively. This trend was true for spheroids up to diameters of ~1,000 μm. In larger spheroids the correlation between glucose supply and central $P_{O_2}$ was reversed. In general, there was a tendency toward increasing central $P_{O_2}$ values with increasing size in large spheroids under all these culture conditions. Spheroids cultured in 0.8 mM glucose exhibited a large variability in central $P_{O_2}$ as a function of size when they were grown to diameters >900 μm. The differences in the size ranges investigated are mainly due to the fact that spheroids in low glucose concentrations could not be grown to diameters as large as those obtained in high glucose concentrations.

The central $P_{O_2}$ values in spheroids cultured in 16.5 mM were also lower than those in spheroids grown under standard conditions, if spheroids <1,000 μm in diameter are considered (see Figure 2). In spheroids >1,000 μm the central $P_{O_2}$ was slightly higher in medium with high glucose concentrations than in standard medium, yet dropped to 0 mm Hg in very large spheroids.

In Figure 3 the correlation between central $P_{O_2}$ and spheroid size is shown for spheroids grown in media with four different glucose concentrations and with a $P_{O_2}$ in the medium of 40 mm Hg. For comparison, the corresponding correlation in spheroids cultured under standard growth conditions is also plotted in these diagrams. Central $P_{O_2}$ values were at 0 mm Hg or a few millimetres of mercury under all of these conditions except for the small spheroids grown in medium containing 0.8 mM glucose. By comparing Figure 2 and Figure 3 it can be seen that at constant glucose concentrations a decreased $P_{O_2}$ in the mdeium during spheroid growth was associated with a reduction in spheroid oxygenation, as expected. The data in Figure 3 also demonstrate that a reduction of the glucose concentration from 5.5 mM to 0.8 mM in media with a lowered $P_{O_2}$ was paralleled by an elevation of the central $P_{O_2}$ values from 1 to 10 mm Hg if average values for the size range of 300–500 μm were considered. In media containing 0.8 mM glucose at low $P_{O_2}$ values, the spheroids did not reach diameters of 1,000 μm, so that central $P_{O_2}$ values could only be measured over a relatively small range of spheroid sizes.

**Figure 3** Central $P_{O_2}$ values in EMT6/Ro spheroids at different stages of growth cultured under standard growth conditions (●) or in media with four different glucose concentrations (△) 0.8 mM; (□) 1.8 mM; (○) 5.5 mM; (◇) 16.5 mM equilibriated with 5% (v/v) $O_2$.

The findings that lowering or increasing the glucose concentration in media during spheroid growth can lead to a deterioration of spheroid oxygenation, and that lowering glucose concentration may, on the other hand, increase central $P_{O_2}$ values under certain conditions, strongly suggest that the external $O_2$ and glucose supply conditions may alter the oxygenation status of spheroids by at least two different, partially counteracting mechanisms. Apparently, the impact of the external supply conditions on central $P_{O_2}$ values is mediated through influences on: (i) the total number of respiring cells per spheroid; and (ii) the respiratory activity of the individual cells.

To estimate the number of viable cells per spheroid, the thickness of the viable rim was determined in spheroids cultured under all the supply conditions investigated. In general, the thickness of the viable rim was less in spheroids cultured in low glucose concentrations than in those maintained in high glucose concentrations (see Figure 4). This holds for spheroids cultured in media with a standard $P_{O_2}$ as well as with reduced external $O_2$ supply, however, in the latter case there was little change in rim thickness when the glucose concentration was lowered from 1.8 to 0.8 mM. At a given glucose concentration, the viable rim was thinner in media with a low $P_{O_2}$ value than in those equilibrated with air. However, in media containing 16.5 mM glucose this correlation is reversed ($P < 0.05$).

**Figure 4** Thickness of the viable rim (mean ±s.d.) in EMT6/Ro spheroids cultured in media with four different glucose concentrations (▲/△) 0.8 mM; (■/□) 1.8 mM; (●/○) 5.5 mM, (◆/◇) 16.5 mM equilibrated either with 20% (v/v) $O_2$ (closed symbols) or with 5% (v/v) $O_2$ (open symbols). In media with a high $P_{O_2}$ a logarithmic correlation between viable rim thickness ($y$) and glucose concentration ($x$) is obtained: $y = 45.94 \ln x + 123.54$; $r = 0.999$; $0.8 \leq x \leq 16.5$; $n = 15$–$25$.

**Figure 5** Volume-related $O_2$ consumption rates $Q$ (mean ± s.e.) in the viable rim of EMT6/Ro spheroids cultured in media with four different glucose concentrations (▲/△) 0.8 mM; (■/□) 1.8 mM; (●/○) 5.5 mM; (◆/◇) 16.5 mM equilibrated either with 20% (v/v) $O_2$ (closed symbols) or with 5% (v/v) $O_2$ (open symbols). Logarithmic correlations among $Q(y)$ and glucose concentrations ($x$) were obtained in media with high $P_{O_2}$ ($y = -1.379 \ln x + 5.491$, $r = 0.997$, $0.8 \leq x \leq 5.5$) and in media with low $P_{O_2}$ ($y = -1.453 \ln x + 4.179$, $r = 0.999$, $0.8 \leq x \leq 5.5$).

Knowing the volume of viable cells (see Figure 4), the $O_2$ consumption rate per volume of 'viable tissue' ($Q$) can be derived from the $P_{O_2}$ gradient in the diffusion-depleted zone as mentioned before. The results of this procedure are demonstrated in Figure 5 for all the growth conditions investigated. Compared to standard growth conditions, lowering of the glucose concentration in the growth media leads to an increase in $Q$. For example, when the glucose concentration was lowered from 5.5 to 0.8 mM, the mean $Q$ values was elevated from 3.09 to $5.73 \times 10^{-4} cm^3_{O_2} \cdot cm^{-3} \cdot s^{-1}$.

A similar increase in $Q$ was observed during the same change in glucose concentration but using growth media with a lowered $P_{O_2}$. At a given glucose concentration, a decrease in the external $P_{O_2}$ was paralleled by a considerable reduction in $Q$, which is true for all glucose concentrations investigated. For example, the average $Q$ value was reduced from 3.09 to $1.65 \times 10^{-4} cm^3_{O_2} \cdot cm^{-3} \cdot s^{-1}$ when the $P_{O_2}$ in media containing 5.5 mM glucose was changed from 145 mmHg to ~40 mmHg (see Figure 5). An increase in the glucose concentration from 5.5 to 16.5 mM did not induce any substantial changes in $Q$ in spheroids cultured in media with either low or high $P_{O_2}$ values. The small differences observed in media with a reduced $P_{O_2}$ (see Figure 5) was not statistically significant ($P > 0.05$).

The data shown in Figure 5 represent the mean (±s.e.) volume-related $O_2$ consumption rates in populations of spheroids of a wide range of sizes. Considering the $Q$ values of each individual spheroid measured independently a trend of changes in $Q$ with spheroid size could be observed; this is demonstrated in Figure 6 for the eight different culturing conditions investigated. Despite a considerable scatter in the data, there is evidence for a decrease in $Q$ with increasing spheroid diameter in at least six out of eight different growth conditions. In addition, the $Q$ values indicate that this tendency may be particularly pronounced in the size range containing the smallest spheroids.

**Figure 6** Volume-related $O_2$ consumption rates $Q$ in the viable rim of individual EMT6/Ro spheroids at different stages of growth. (a) Spheroids cultured in media with 5.5 mM (●/○) or 16.5 mM glucose (◆/◇) equilibrated with 20% (v/v) $O_2$ (closed symbols) or 5% (v/v) $O_2$ (open symbols). (b) Spheroids cultured in media with 1.8 mM (■/□) or 0.8 mM glucose (▲/△) equilibrated with 20% (v/v) $O_2$ (closed symbols) or 5% (v/v) $O_2$ (open symbols).

The scatter in the data is more pronounced at a glucose concentration of 0.8 mM than at higher glucose concentrations. The variability in central $P_{O_2}$ mentioned before under the respective growth conditions, may therefore be mainly attributed to a variability in the respiration rate of individual spheroids cultured under the same conditions.

The $O_2$ diffusion conductivity ($K_s$) in the spheroids, which was derived from the $P_{O_2}$ gradient in the viable zones of the spheroids (Mueller-Klieser, 1984), was essentially the same under all growth conditions investigated being 1.85–1.89 × $10^{-5}$ $cm^3_{O_2} \cdot cm^{-1} min^{-1} \cdot atm^{-1}$. This value is in good agreement with the values found in solid tumours (Grote *et al.*, 1977). The values obtained for $K_s$ and $Q$ and the data from histological investigation on the thickness of the viable rims allow the theoretical calculation of $P_{O_2}$ profiles that are in accordance with the $P_{O_2}$ distributions measured (see Figure 1).

### Discussion

The results of the present investigation clearly show that there is a large influence of both the external glucose and $O_2$ supply during spheroid growth on the oxygenation status of tumour cells in multicellular spheroids. These changes are mediated through an impact of the glucose concentration and of the $O_2$ tension in the growth media on both the cellular viability and on the volume-related $O_2$ consumption rate ($Q$) in the viable area of the spheroid. Since significant changes in cellular packing density during spheroid growth have not been observed in EMT6-spheroids (Freyer & Sutherland, 1985*c*), the changes in $Q$ probably results from variations in the respiratory activity of the cells.

In the present study, the tumour cells are continuously exposed to different external glucose concentrations for a period of several weeks. This leads to persistent changes in $Q$ which are qualitatively similar to those observed by Crabtree (1929), i.e. an increase in the extracellular glucose concentration is followed by a decrease in $O_2$ consumption at least within a certain range of glucose concentrations (see Figure 5). However, those changes in cellular respiration, which are commonly referred to as the Crabtree effect, are elicited by acute changes in the external glucose supply. In contrast to the present observation, the Crabtree effect is a transient phenomenon with a variation in respiration that is much smaller than the changes in $Q$ shown in Figure 5. To our knowledge changes in the oxygen consumption rate of tumour cells suggesting a chronic adaptation of metabolism to varying external glucose supply conditions have not been described previously. This adaptation of metabolism may also occur in tumour cells *in vivo* and may, thus, be relevant for tumour growth and susceptibility to treatment.

The chronic changes in $Q$ as a function of the extracellular glucose concentration exhibit a saturation characteristic above glucose concentrations of 5.5 mM. $Q$ values are not significantly different in spheroids grown in 16.5 mM compared to 5.5 mM glucose. Obviously, there is no adaptation of cellular respiration to external glucose concentrations considerably above the normal plasma concentration.

A chronic decrease in the external $O_2$ supply leads to a persistent reduction of the $O_2$ consumption in spheroids at all glucose concentrations applied. Although the central $P_{O_2}$ values are close to 0 mmHg under almost all of these culturing conditions (see Figure 3), the $P_{O_2}$ gradients are steep throughout the viable rim of the respective spheroids. This finding indicates the absence of any substantial reduction in $Q$ in the inner parts *versus* the outer parts of these spheroids. A possible reduction in $Q$ as a consequence of lowered but still suffcient $O_2$ supply would be associated with an increase in the $O_2$ diffusion length and would, therefore, be of great significance for tumour growth *in vivo*.

Spheroid size may also influence the $O_2$ consumption rate (see Figure 6). A large change in $Q$, as a function of spheroid size, has been found recently by Freyer & Sutherland (1984) in spheroids from V79 cells. The $O_2$ consumption rate in very small V79 spheroids was similar to that of single cells, yet dropped to approximately one third to one fourth of that value when spheroids grew from diameters of 200 $\mu$m up to 400 $\mu$m. Little further decrease in $Q$ was observed beyond that stage of growth. The data presented here suggest that considerable changes in $Q$ may also occur in EMT6 spheroids during growth, but these changes may be distributed over a wider diameter range than in the case of V79 spheroids. This is confirmed by recent observations in EMT6 spheroids cultured under standard growth conditions (Freyer & Sutherland, 1985*c*; Mueller-Klieser *et al.*, 1986). Since similar changes may be true for all the culture conditions investigated (see Figure 6), changes in $Q$ during spheroid growth may, therefore, not be a result of one specific supply situation but rather a general characteristic of three-dimensional growth of tumour cells.

The method for the determination of $Q$ used in this study does not account for local variations in $Q$ within the viable rim of spheroids. Such variations may occur due to changes in the proliferative status and/or in the cell packing density in spheroids (Grossmann *et al.*, 1984; Sutherland *et al.*, 1985). Since almost all of the experimental $P_{O_2}$ profiles recorded in this investigation can be sufficiently approximated using constant $Q$ values (e.g. see Figure 1), it is concluded that only minor local changes in $Q$ may be present in EMT6-spheroids even when cultured under different supply conditions. This is surprising, because proliferative gradients have been found previously in EMT6 spheroids (Freyer & Sutherland, 1980), so that one would expect a non-uniform $O_2$ consumption across the viable rim of these spheroids. A factor that may counteract the reduction of $Q$ in the inner part *versus* the outer part of spheroids may be a decreasing glucose concentration with increasing distance into the spheroid. Also, cell-cell interaction may have an influence on $Q$, e.g. by modifying the inter-relationship among $Q$ and the proliferative status of the cells. In a recent study, the authors have found significant local variations of $Q$ in spheroids from a human colon carcinoma using similar techniques as in the present investigation (Sutherland *et al.*, 1986). Thus, the data available at present suggest that local variations in $Q$ may be an intrinsic characteristic of the cell line used and they may depend on the culturing technique used for spheroid growth.

An impact of the $O_2$ tension in the growth media on the thickness of the viable rim in V79 spheroids has been demonstrated in previous investigations (Franko & Sutherland, 1979*a*). With the exception of media with high glucose concentration, the present data from EMT6 spheroids confirm those earlier findings, since a decrease in the external $O_2$ tension leads to a reduction in the viable rim thickness (see Figure 4). In addition, the present data demonstrate that the viable rim thickness is strongly dependent on the external glucose concentration both for low and high external $O_2$ tensions (see Figure 4).

In general, there is a deterioration of the spheroid oxygenation during spheroid growth as indicated by a decrease in the central $P_{O_2}$ with increasing spheroid diameter (see Figures 2 and 3). The central $P_{O_2}$ decreases from very high values ($\gg$0 mm Hg) towards minimum values of 0 or a few mm Hg, and then stays at this level or increases again with further spheroid growth. Diffusion calculations and histological observations indicate that the increase in the central $P_{O_2}$ in larger spheroids is mainly due to a decrease in the thickness of the viable cell rim, i.e., by a reduction in the number of respiring cells at a given spheroid size (Freyer & Sutherland, 1985*a,b*). The diameter at which the minimum central $P_{O_2}$ is reached appears to be strongly dependent on the external $O_2$ and glucose supply during spheroid growth. Thus, the inter-relationship among central $P_{O_2}$ and spheroid size may show a similar pattern under all growth conditions investigated, yet the decrease of the central $P_{O_2}$ with increasing spheroid size has not been detected under some of these conditions since the minimum central $P_{O_2}$ has been already reached in very small spheroids not suited for micro-electrode measurements (see Figures 2 and 3).

There is evidence from the present data, from data published previously (Carlsson *et al.*, 1979; Mueller-Klieser & Sutherland, 1982*a,b*; Mueller-Klieser *et al.*, 1983) as well as from theoretical considerations (Mueller-Klieser, 1984; Freyer & Sutherland, 1985*a,b*) that necrosis in spheroids may develop despite sufficient $O_2$ supply. This is true for most of the supply conditions investigated, yet in media with 5.5 mM glucose and a $P_{O_2}$ of 35–40 mm Hg the central $P_{O_2}$ had dropped to 0 mm Hg before necrosis arises in the spheroid centre.

Therefore, lack of oxygen may induce cell death in spheroid centres under those conditions which closely approximate the supply conditions of cells in solid tumours.

It can be shown by theoretical considerations that limitation of glucose supply may also be the cause of the development of necrosis in some of the growth conditions investigated; however, necrosis can occur despite sufficient $O_2$ and glucose supply (Mueller-Klieser *et al.*, 1983; Freyer & Sutherland, 1985*a,b*; Mueller-Klieser, 1985). Factors other than $O_2$ and glucose depletion that are partially still unknown may therefore be involved in the development of necrosis at least in spheroids cultured under specific supply conditions. The accumulation of metabolic waste products such as $H^+$ ions, lactate or ammonia may play a significant role in causing cell death. On the other hand, it is also possible that products from cell lysis in necrotic areas may also influence cellular viability, proliferation and metabolism. In general, spheroids may not only be considered diffusion-limited tissue models with regard to diffusion of $O_2$ and nutrient *into* the aggregates. Limitation of the *outward* diffusion of metabolic waste products may be of equal or even higher relevance for the biolgoical behaviour of the cancer cells in these aggregates.

The data obtained in the present investigation show that cell death in tumours may be the result of interaction of several mechanisms and may be more complex than seen from the 'classical' point of view of only $O_2$ diffusion limiation as postulated by Thomlinson & Gray (1955). The capacity observed of tumours cells to adapt their proliferative and metabolic status to various environmental situations impedes predictions on diffusion distances in tumours *in vivo*. The findings make it evident that more research should be done to further elucidate pathophysiological mechanisms determining cellular viability, proliferation and metabolism in solid tumours.

This research was supported by NIH grants CA-20239, CA-11198, and CA-11051, by grants Mu 576/1 and Mu 576/2-1 from the Deutsche Forschungsgemeinschaft and by the Alexander von Humboldt Foundation through a Senior U.S. Scientist Award for Robert M. Sutherland. The investigations were also performed under DOE contract number DE-AC02-7 6EV03490. The technical assitance was provided by Gertrude Nielsen and Betty Bareham. Histological services were provided by the Experimental Pathology/Ultrastructure Facility of the University of Rochester Cancer Center.

### References

ANGELLO, J.C. & HOSICK, H.L. (1982). Glycoaminoglycan synthesis by mammary tumor spheroids. *Biochem. Biophys. Res. Commun.*, **107**, 1130.

BALIN, A., GOODMAN, D.B.P., RASMUSSEN, H. & CRISTOFALO, V.J. (1976). The effect of oxygen tension on the growth and metabolism of WI-38 cells. *J. Cell. Physiol.*, **89**, 235.

CARLSSON, J., STALNACKE, G., ACKER, H., HAJI-KARIM, M., NILSSON, S. & LARSSON, B. (1979). The influence of oxygen on viability and proliferation in cellular spheroids. *Int. J. Radiat. Oncol. Biol. Phys.*, **5**, 2011.

CECCARINI, C. & EAGLE, H. (1971). pH as a determinant of cellular growth and contact inhibition. *Proc. Natl Acad. Sci. (USA)*, **68**, 229.

CRABTREET, H.H. (1929). LXI. Observations on the carbohydrate metabolism of tumours. *Biochem. J.*, **23**, 536.

DERTINGER, H. & HÜLSER, D. (1981). Increased radioresistance of cells in cultured multicell spheroids. I. Dependence on cellular interaction. *Radiat. Environ. Biophys.*, **19**, 101.

FRANKO, A.J. & SUTHERLAND, R.M. (1979*a*). Oxygen diffusion distance and development of necrosis in multicell spheroids. *Radiat. Res.*, **79**, 439.

FRANKO, A.J. & SUTHERLAND, R.M. (1979*b*). Radiation survival of cells from spheroids grown in different oxygen concentrations. *Radiat. Res.*, **79**, 454.

FREYER, J.P. & SUTHERLAND, R.M. (1980). Selective dissociation and characterization of cells from different regions of multicell tumor spheroids. *Cancer Res.*, **40**, 3956.

FREYER, J.P. & SUTHERLAND, R.M. (1984). *In situ* oxygen consumption rates of cells in V-79 multicellular spheroids during growth. *J. Cell. Physiol.*, **118**, 53.

FREYER, J.P. & SUTHERLAND, R.M. (1985*a*). Regulation of growth, viability and cell subpopulations in multicell spheroids by oxygen and glucose. I. Spheroid growth rates and development of necrosis. *Cancer Res.* (in press).

FREYER, J.P. & SUTHERLAND, R.M. (1985*b*). Regulation of growth, viability and cell subpopulations in multicell spheroids by oxygen and glucose. II. Cell cycle kinetic and cell clonogenicity. *Cancer Res.* (in press).

FREYER, J.P. & SUTHERLAND, R.M. (1985*c*). A reduction in the *in situ* rates of oxygen and glucose consumption of cells in EMT6/Ro spheroids during growth. *J. Cell. Physiol.*, **124**, 516.

GROSSMANN, U., WINKLER, P., CARLSSON, J. & ACKER, H. (1984). Local variations of oxygen consumption within multicellular spheroids calculated from $P_{O_2}$ profiles. *Adv. Exp. Med. Biol.*, **169**, 719.

GROTE, J., SUESSKIND, R. & VAUPEL, P. (1977). Oxygen diffusivity in tumor tissue (DS-Carcinosarcoma) under temperature conditions within the range of 20–40°C. *Pflügers Arch.*, **372**, 37.

GUPTA, V. & EBERLE, R. (1984). Modulation of tumor cell colony growth in soft agar by oxygen and its mechanism. *Br. J. Cancer*, **49**, 587.

MUELLER-KLIESER, W. (1984). Method for the determination of oxygen consumption rates and diffusion coefficient in multicellular spheroids. *Biophys. J.*, **46**, 343.

MUELLER-KLIESER, W. (1985). Limitierende Faktoren für die Versorgung von Tumorgeweben – Experimentelle und theoretische Untersuchungen am Spharoidmodell. In *Funktionsanalyse biologischer Systeme,* Bd. 13, Thews, G. (ed), Steiner Verlag: Wiesbaden.

MUELLER-KLIESER, W. & SUTHERLAND, R.M. (1982*a*). Influence of convection in the growth medium on oxygen tensions in multicellular tumor spheroids. *Cancer Res.,* **42,** 237.

MUELLER-KLIESER, W. & SUTHERLAND, R.M. (1982*b*). Oxygen tensions in multicellular spheroids of two cell lines. *Br. J. Cancer,* **45,** 256.

MUELLER-KLIESER, W., FREYER, J.P. & SUTHERLAND, R.M. (1983). Evidence for a major role of glucose in controlling development of necrosis in EMT6/Ro multicell tumor spheroids. *Adv. Exp. Med. Biol.,* **159,** 487.

MUELLER-KLIESER, W., BOURRAT, B., GABBERT, H. & SUTHERLAND, R.M. (1986). Changes in $O_2$-consumption of multicellular spheroids during development of necrosis. *Adv. Exp. Med. Biol.* (in press).

NEDERMAN, T., NARLING, B., GLIMELIUS, B., CARLSSON, J. & BRUNK, U. (1984). Demonstration of an extracellular matrix in multicellular tumor spheroids. *Cancer Res.,* **44,** 3090.

POSTE, G. & GREIG, R. (1983). The experimental and clinical implications of cellular heterogeneity in malignant tumors. *J. Cancer Res. Clin. Oncol.,* **106,** 159.

ROCKWELL, S.C., KALLMAN, R.F. & FAJARDO, L.F. (1972). Characteristics of a serially-transplanted mouse mammary tumor and its tissue culture adapted derivative. *J. Natl Cancer Inst.,* **49,** 735.

SUTHERLAND, R.M. & DURAND, R.E. (1976). Radiation response of multicellular spheroids – an *in vitro* tumor model. *Curr. Top. Radiat. Res.,* **11,** 87.

SUTHERLAND, R.M., SORDAT, B., GABBERT, H., BOURRAT, B. & MUELLER-KLIESER, W. (1986). Oxygen tension measurements in multicellular spheroids derived from two different human colon carcinomas. *Cancer Res.* (in press).

THOMLINSON, R.H. & GRAY, L.H. (1955). The histological structure of some human lung cancers and the possible implications for radiotherapy. *Br. J. Cancer,* **9,** 539.

VAUPEL, P., FRINAK, S. & BICHER, H. (1981). Heterogeneous oxygen partial pressure and pH distribution in C3H mouse mammary adenocarcinoma. *Cancer Res.,* **41,** 2008.

WIGLE, J.C., FREYER, J.P. & SUTHERLAND, R.M. (1983). Use of a sedimentation column to obtain uniformly sized populations of multicell spheroids. *In vitro,* **19,** 361.